# Supplementary figures and images for: Construction of a reference transcriptome for the analysis of male sterility in sugi (Cryptomeria japonica D. Don) focusing on MALE STERILITY 1 (MS1)
Source: PLoS One. 2021 Feb 25;16(2):e0247180. doi: 10.1371/journal.pone.0247180 (PMC7935350; doi:10.1371/journal.pone.0247180)

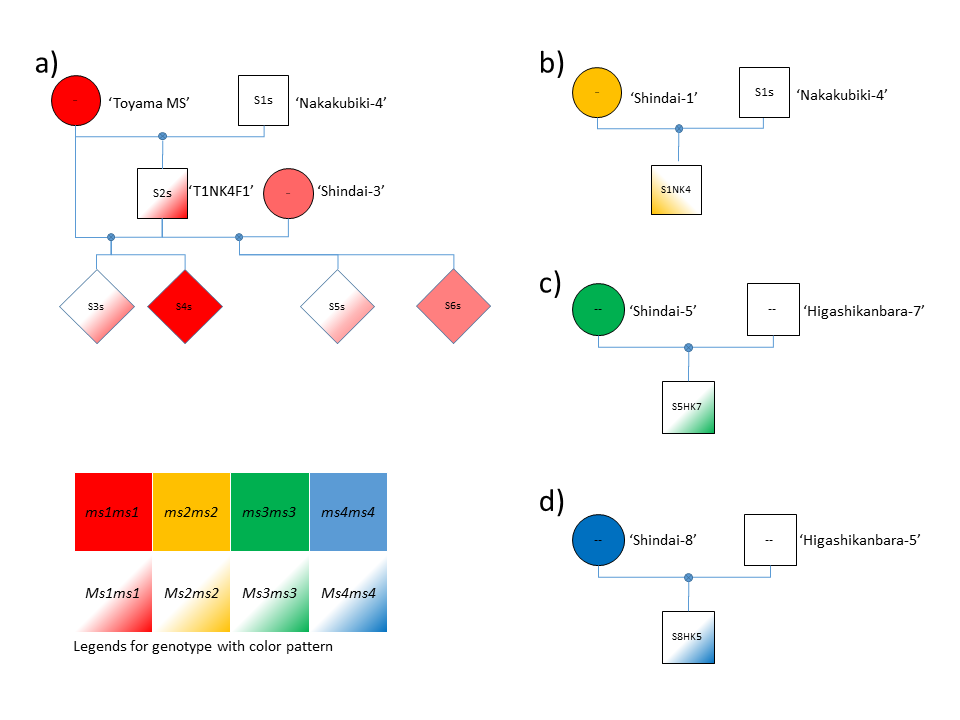

Supplement: S1 Fig — Pedigrees for the analysis of a) MS1, b) MS2, c) MS3, and d) MS4. Genotypes for male sterility genes are indicated by color patterns, where solid colors and gradient colors correspond to male-fertile and male-sterile genotypes, respectively. Inside shapes are the names of RNA-Seq library working IDs (Table 1). (PNG) [file pone.0247180.s013.png]

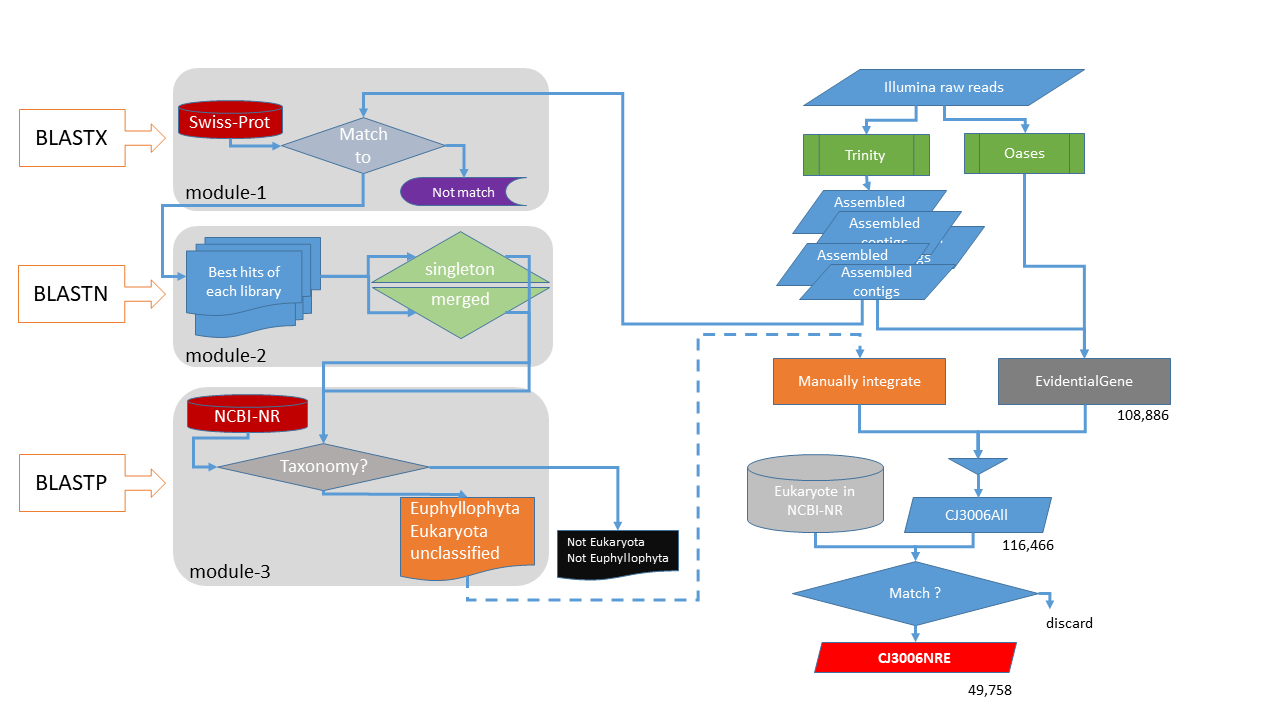

Supplement: S2 Fig — The general workflow of assembly and the workflow of the semi-manual assembly (module-1, module-2, and modle-3). (PNG) [file pone.0247180.s014.png]

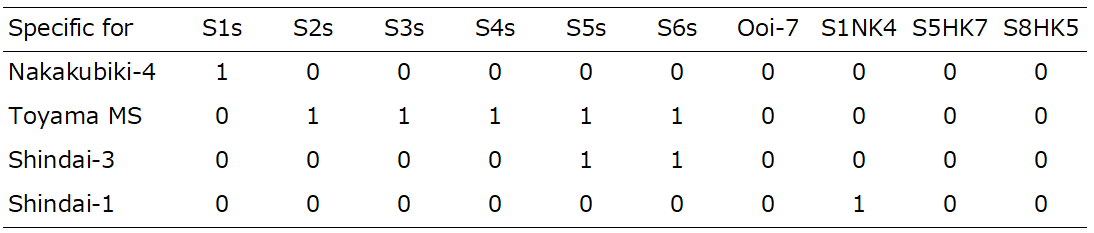

Supplement: S3 Fig — This bitmap pattern was used in the “bcftools isec” command to select group specific variants. (PNG) [file pone.0247180.s015.png]

## Slide 1
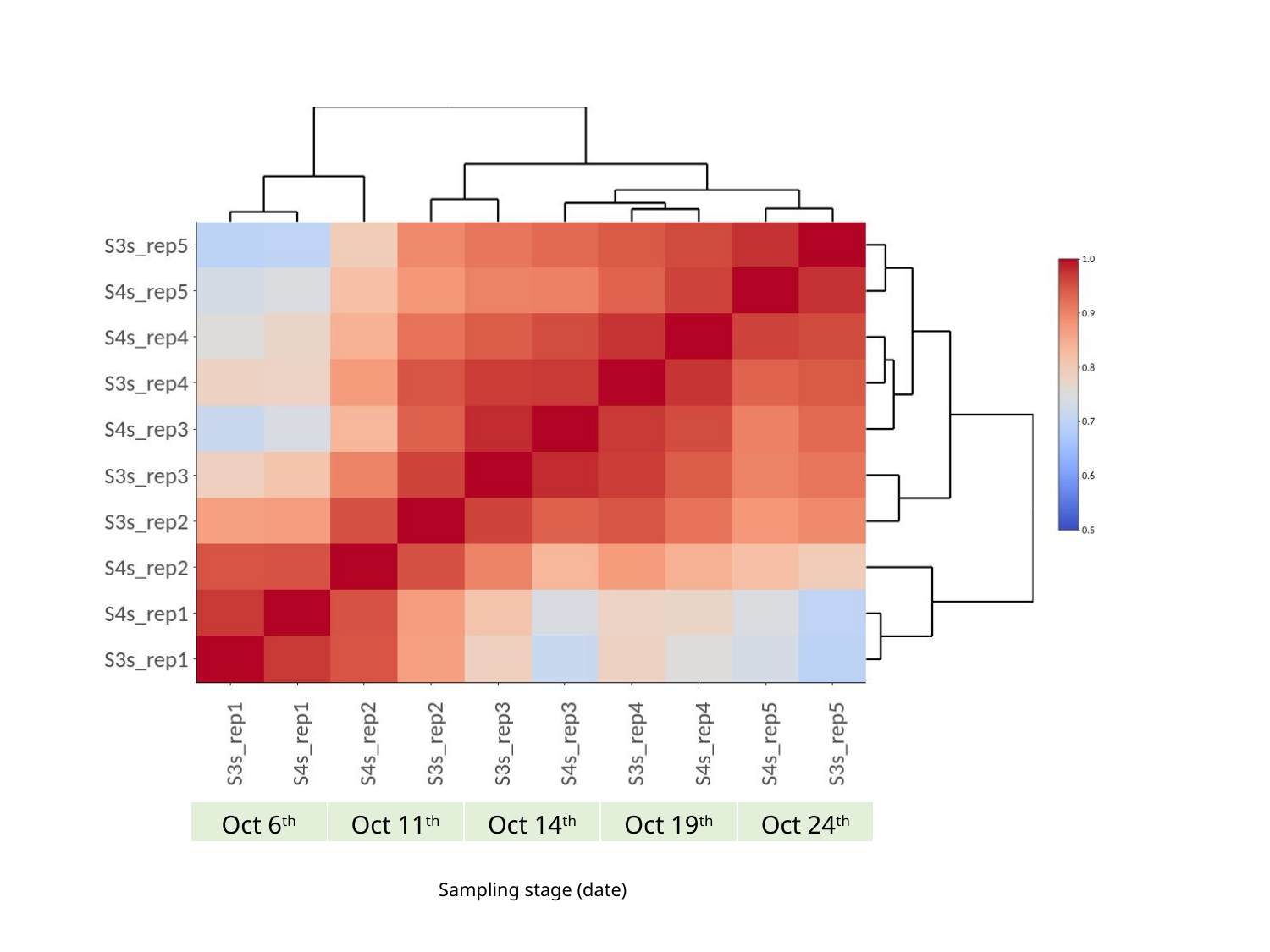

| Oct 6th | Oct 11th | Oct 14th | Oct 19th | Oct 24th |
| --- | --- | --- | --- | --- |
Sampling stage (date)

Supplement: S6 Fig — Correlations were analyzed via a time-series of sampling, dated from 6th Oct to 24th Oct. (PPTX) [file pone.0247180.s018.pptx]
