# Supplementary material for: Construction of a reference transcriptome for the analysis of male sterility in sugi (Cryptomeria japonica D. Don) focusing on MALE STERILITY 1 (MS1)
Source: PLoS One. 2021 Feb 25;16(2):e0247180. doi: 10.1371/journal.pone.0247180 (PMC7935350; doi:10.1371/journal.pone.0247180)
Supplement: S4 Fig — Each dot indicates a pairwise comparison of RNA-Seq libraries. MF: Male flower, IBL: inner bark and leaf, MFIBL: male flower, inner bark, and leaf. (PPTX) [file pone.0247180.s016.pptx]

## Slide 1
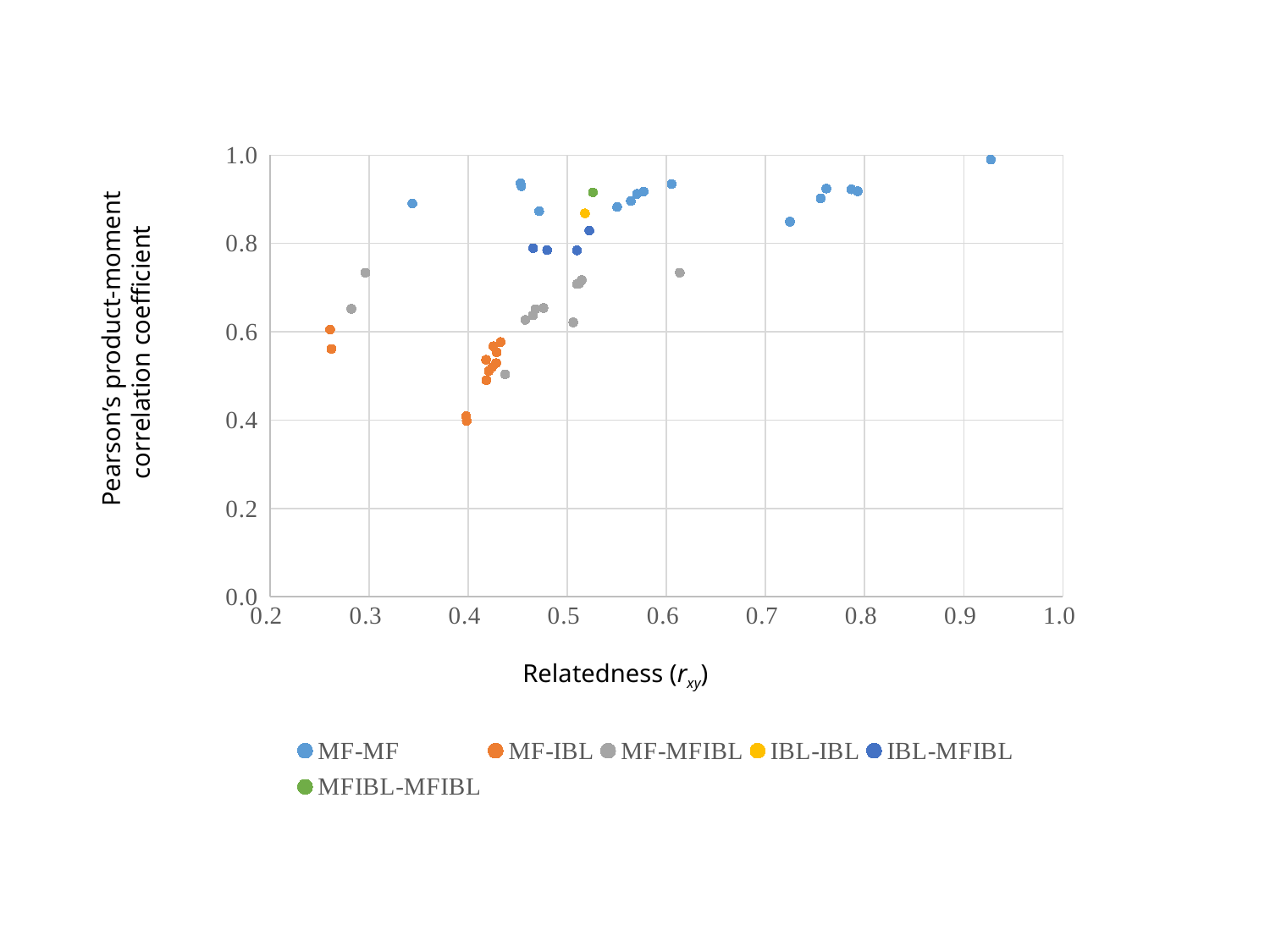

### Chart
| Category | MF-MF | MF-IBL | MF-MFIBL | IBL-IBL | IBL-MFIBL | MFIBL-MFIBL |
|---|---|---|---|---|---|---| Pearson’s product-moment
correlation coefficient
Relatedness (rxy)
