# Supplementary material for: Construction of a reference transcriptome for the analysis of male sterility in sugi (Cryptomeria japonica D. Don) focusing on MALE STERILITY 1 (MS1)
Source: PLoS One. 2021 Feb 25;16(2):e0247180. doi: 10.1371/journal.pone.0247180 (PMC7935350; doi:10.1371/journal.pone.0247180)
Supplement: S5 Fig — The x-axis indicates the number of read pairs in each library, while the y-axis indicates BUSCO results ratios for Complete, Duplicated, Fragments, Missing, and Singles. (PDF) [file pone.0247180.s017.pdf]

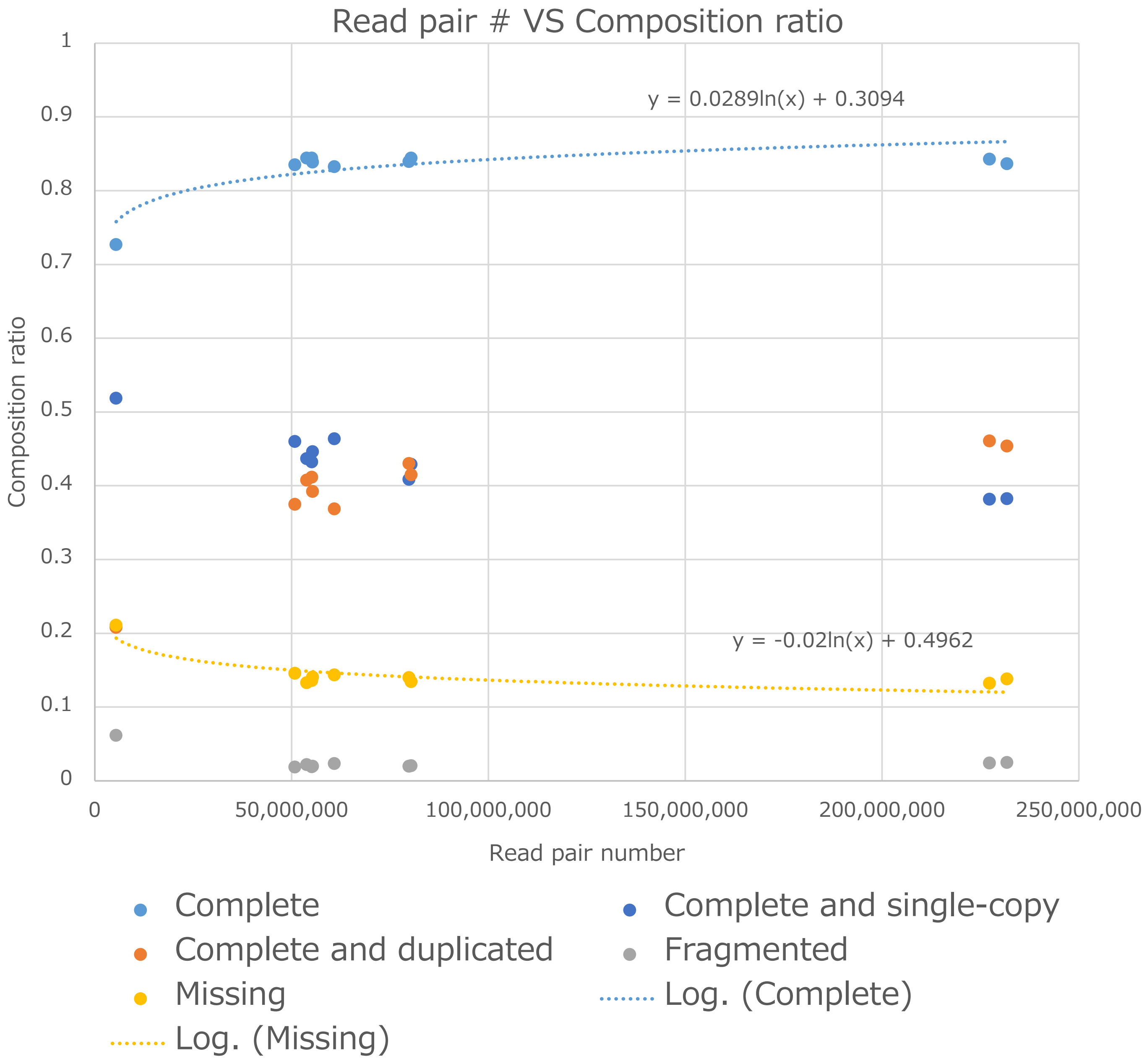

Read pair # VS Composition ratio
y = 0.0289ln(x) + 0.3094
y = -0.02ln(x) + 0.4962
Composition ratio
1
0.9
0.8
0.7
0.6
0.5
0.4
0.3
0.2
0.1
0
0
50,000,000
100,000,000
150,000,000
200,000,000
250,000,000
Read pair number
Complete
Complete and single-copy
Complete and duplicated
Fragmented
Missing
Log. (Complete)
Log. (Missing)
