# Supplementary material for: Construction of a reference transcriptome for the analysis of male sterility in sugi (Cryptomeria japonica D. Don) focusing on MALE STERILITY 1 (MS1)
Source: PLoS One. 2021 Feb 25;16(2):e0247180. doi: 10.1371/journal.pone.0247180 (PMC7935350; doi:10.1371/journal.pone.0247180)
Supplement: S7 Fig — Microscopic images (400x) of pollen, taken on 19th Oct. 2011, for S3s and S4s are embedded in the graph. (PPTX) [file pone.0247180.s019.pptx]

## Slide 1
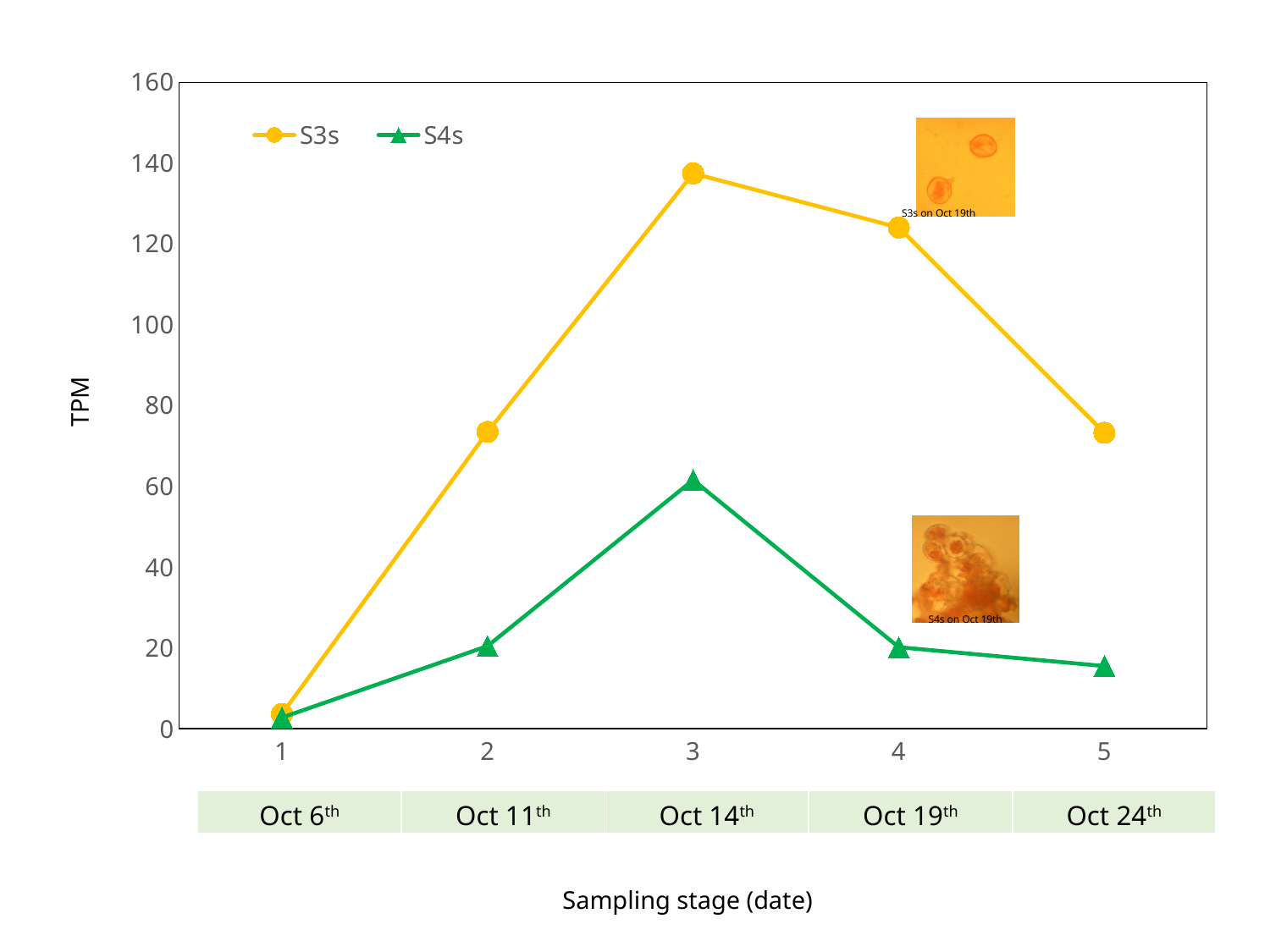

### Chart
| Category | S3s | S4s |
|---|---|---|
S3s on Oct 19th
TPM
S4s on Oct 19th
| Oct 6th | Oct 11th | Oct 14th | Oct 19th | Oct 24th |
| --- | --- | --- | --- | --- |
Sampling stage (date)
